# Intrauterine *Gardnerella vaginalis* Infection Results in Fetal Growth Restriction and Alveolar Septal Hypertrophy in a Rabbit Model

**DOI:** 10.3389/fped.2020.593802

**Published:** 2021-01-22

**Authors:** Fook-Choe Cheah, Chee Hoe Lai, Geok Chin Tan, Anushia Swaminathan, Kon Ken Wong, Yin Ping Wong, Tian-Lee Tan

**Affiliations:** ^1^Department of Pediatrics, Universiti Kebangsaan Malaysia Medical Center, Kuala Lumpur, Malaysia; ^2^Department of Pathology, Universiti Kebangsaan Malaysia Medical Center, Kuala Lumpur, Malaysia; ^3^Department of Microbiology, Universiti Kebangsaan Malaysia Medical Center, Kuala Lumpur, Malaysia

**Keywords:** bacterial vaginosis, intrauterine growth restriction, bronchopulmonary dysplasia, intra-amniotic, rabbit model, multinucleated syncytiotrophoblasts, fetal lung development, intrauterine infection

## Abstract

**Background:** *Gardnerella vaginalis* (GV) is most frequently associated with bacterial vaginosis and is the second most common etiology causing intrauterine infection after *Ureaplasma urealyticum*. Intrauterine GV infection adversely affects pregnancy outcomes, resulting in preterm birth, fetal growth restriction, and neonatal pneumonia. The knowledge of how GV exerts its effects is limited. We developed an *in vivo* animal model to study its effects on fetal development.

**Materials and Methods:** A survival mini-laparotomy was conducted on New Zealand rabbits on gestational day 21 (28 weeks of human pregnancy). In each dam, fetuses in the right uterine horn received intra-amniotic 0.5 × 10^2^ colony-forming units of GV injections each, while their littermate controls in the left horn received sterile saline injections. A second laparotomy was performed seven days later. Assessment of the fetal pups, histopathology of the placenta and histomorphometric examination of the fetal lung tissues was done.

**Results:** Three dams with a combined total of 12 fetuses were exposed to intra-amniotic GV, and 9 fetuses were unexposed. The weights of fetuses, placenta, and fetal lung were significantly lower in the GV group than the saline-inoculated control group [mean gross weight, GV (19.8 ± 3.8 g) vs. control (27.9 ± 1.7 g), *p* < 0.001; mean placenta weight, GV (5.5 ± 1.0 g) vs. control (6.5 ± 0.7 g), *p* = 0.027; mean fetal lung weight, GV (0.59 ± 0.11 g) vs. control (0.91 ± 0.08 g), *p* = 0.002. There was a two-fold increase in the multinucleated syncytiotrophoblasts in the placenta of the GV group than their littermate controls (82.9 ± 14.9 vs. 41.6 ± 13.4, *p* < 0.001). The mean alveolar septae of GV fetuses was significantly thicker than the control (14.8 ± 2.8 μm vs. 12.4 ± 3.8 μm, *p* = 0.007). Correspondingly, the proliferative index in the interalveolar septum was 1.8-fold higher in the GV group than controls (24.9 ± 6.6% vs. 14.2 ± 2.9%, *p* = 0.011). The number of alveoli and alveolar surface area did not vary between groups.

**Discussion:** Low-dose intra-amniotic GV injection induces fetal growth restriction, increased placental multinucleated syncytiotrophoblasts and fetal lung re-modeling characterized by alveolar septal hypertrophy with cellular proliferative changes.

**Conclusion:** This intra-amniotic model could be utilized in future studies to elucidate the acute and chronic effects of GV intrauterine infections.

## Introduction

*Gardnerella vaginalis* (GV) is the first bacterium found to play a significant role in bacterial vaginosis (BV), which is a dysbiosis of the vaginal microflora that affects 4.7–58.3% of the female population worldwide, particularly in females of reproductive age (1, 2). The majority of women who harbor GV frequently remain untreated unless they developed severe symptoms (3, 4). Therefore, a significant proportion of women may carry this organism while they are pregnant. The high recurrence rate further complicates the course of GV infection (5).

GV infection during the perinatal period is becoming increasingly important as it is associated with unfavorable outcomes for both the mother and growing fetus (2). It is the second most common etiology of intrauterine infection (6–8) and is associated with an increased risk of preterm birth, spontaneous abortion, and maternal infection (9, 10). Postnatally, several neonatal cases of invasive GV infection causing pneumonia, meningitis, osteomyelitis, septicemia, and death had been reported (11–16).

Besides, GV-associated BV served as an independent risk factor for low in birthweight infants and was responsible for as much as 56% of pregnancies complicated with intrauterine growth restriction (IUGR) (17). Some potential mechanisms which could limit the intrauterine fetal growth include placenta dysfunction, thrombosis of the placenta vessels, and tissue necrosis (18). In humans, a prominent histopathological feature in the placenta of IUGR infant is the increase in syncytial knots (19, 20). The syncytial knot (SK) is characterized by a clustering of five or more syncytiotrophoblast nuclei that bulge on top of the normal villous surface. It is presently uncertain whether intrauterine GV infection may be associated with a similar histological change that may explain the underlying cause of IUGR in such cases.

Intrauterine infection also has an impact on the developing fetal lung (21–23). In animal models, the intra-amniotic injection of *Ureaplasma urealyticum, Escherichia coli*, and bacterial lipopolysaccharide were associated with histomorphometric lung changes that resemble bronchopulmonary dysplasia (BPD) (24–26). BPD is a chronic lung disease which primarily affects preterm infants and results in significant morbidity and mortality (27). A recent systematic review concluded that exposure to intrauterine inflammation is associated with a higher risk of developing BPD (28). Nevertheless, the impact of intrauterine GV infection on the development of the fetal lung remains unclear.

Various animal models of intrauterine infection/inflammation have been created to study the effects of pathogens/toxins on the developing fetus (10, 24). In this study, we developed a rabbit model by directly inoculating GV into the amniotic cavity to study its effects on the developing fetus. As opposed to intravaginal inoculation, the direct intra-amniotic route overcomes the issue of vaginal flora variation across species (29–31). Besides, studies have shown that the direct intra-amniotic inoculation of bacteria produces more apparent effects as compared to the deposition of bacteria into the placenta-decidual interface (32–34). Titration of the doses and approach used are crucial to study a specific pathology and reduce attrition from overwhelming infection causing animal deaths (10). As such, we introduced the lowest dose of GV to ensure survival and studied the pathological effects to the fetus after the insult and clearance of the infection.

The female rabbit has a bicornuate duplex uterus, characterized by two completely separated uterine horns with a cervix each. This unique anatomy allows fetuses in one horn to be used as the control littermates for the other horn's as intervention. The littermate-controlled experimental design ensures the standardization of genetic profile, environmental background, and intrauterine condition.

## Materials and Methods

### Animal

Three timed-pregnant New Zealand white rabbits (*Oryctolagus cuniculus*) were obtained from the animal farm of Broga Hill (Selangor, Malaysia), where they were born and bred. They were housed in the experimental facility of the Laboratory Animal Resource Unit (LARU), Universiti Kebangsaan Malaysia under standard care conditions, and fed rabbit chow *ad libitum*. The sample size obtained was 21 (12 in GV-inoculated group; nine in the saline-inoculated group), and based on the “resource equation” calculation formula [degree of freedom, *E* = total number of animal – total number of groups], the *E* = 19 was in the optimal range of 10–20 and this number was considered adequate (35).

The study protocol and sample size were approved by the Universiti Kebangsaan Malaysia Animal Ethics Committee (UKMAEC) (approval code: FP/PAED/2012/CHEAH/26-SEPTEMBER/460-NOVEMBER-2012-NOVEMBER-2014), and all procedures were conducted in compliance with the Malaysian Code of Practice for the Care and Use of Animals for Scientific Purposes. This novel animal model design is registered under the copyright reference number: UKM.IKB.800-4/1/1084.

### Surgical Procedure and Intra-amniotic Inoculation of GV

**Figure 1:**
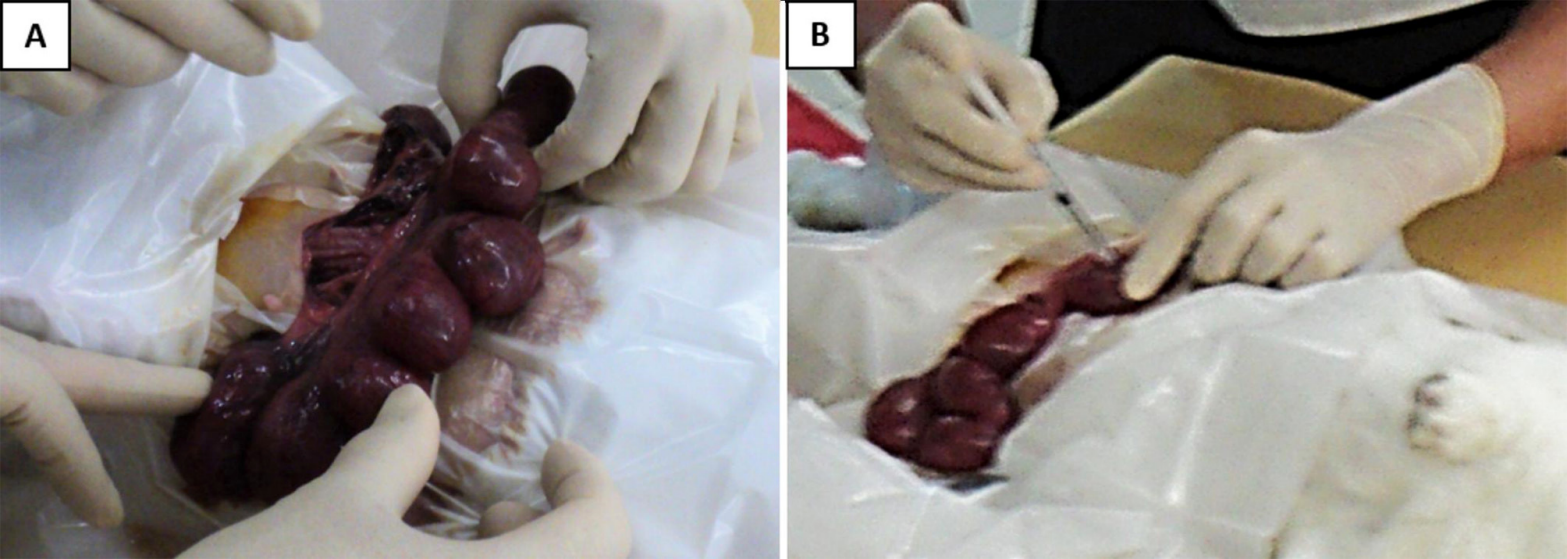
Intra-amniotic injection of GV. **(A)** Following the laparotomy procedure, the gravid uterine horns were externalized and examined. **(B)** Injection was performed directly into the amniotic sac surrounding each of the fetus, with either 0.5 × 10^2^ CFU of GV or sterile saline. The injection was done at a site to avoid hitting the placenta and fetal parts.

The pregnant dams were subjected to a surviving mini-laparotomy surgery on day 21 of gestation (equivalent to 28 weeks human gestation). They were anesthetized with intravenous xylazine 5 mg/kg and ketamine 35 mg/kg via the marginal ear vein, placed supine and prepared in a sterile field. The fur at the surgical area was shaved, and the skin was scrubbed with povidone–iodine. A lower midline mini-laparotomy (incision size: 1–1.5 in.) was then performed, exposing the two uterine horns. The uterine horns were carefully externalized from the abdominal cavity, and the number of viable fetuses in each horn was identified (Figure 1A). The fetuses were assigned to two experimental groups based on the position of the uterine horn. With prefilled standard insulin syringes, the amniotic cavity of fetuses on the right uterine horn was injected with 0.5 mL volume containing GV (10^2^ colony-forming units per mL; ATCC 14018) suspended in Hank's Balanced Salt Solution (HBSS) (Gibco, Massachusetts, United States). As controls, the amniotic cavity of fetuses on the left horn was each injected with 0.5 mL sterile HBSS (Figure 1B). The site of injection was observed for any leakage and sealed with an adhesion spray if necessary. Routine layered abdominal wall closure was performed. Meanwhile, the remaining aliquot of GV suspension was sent to the laboratory to confirm bacterial viability and back-culture quantification.

Upon recovery from the surgery, the dams were housed in individual cages with 12/12 light cycles and *ad libitum* access to a commercially-available pellet diet and sterile water for the next seven days. The animals were examined daily for wound healing and signs of preterm labor.

The dams were subjected to the second laparotomy on day 28 of gestation (equivalent to 37–38 weeks human gestation). Once sedated, the abdominal and uterine walls were sectioned, the fetuses and placenta were removed. The amniotic fluid surrounding each of the fetuses was gently dabbed onto a sterile cotton swab and sent for culture. At the end of the surgery, the dams and fetuses were humanely euthanized with a lethal dose of xylazine-ketamine mixture (2 mL/kg). Each fetus and placenta were weighed individually. The lungs and placenta were harvested and prepared for the various tissue analyses.

### Preparation of GV Suspension

*Gardnerella vaginalis* (ATCC 14018) was used as stock in this model of intrauterine infection. The Gardnerella selective Columbia Blood Agar with sheep blood (Thermo Scientific, Massachusetts, United States) was streaked with GV from the bacteria stock and incubated for 48 h in a 5% CO_2_ atmosphere at 37°C. A 50 mL centrifuge tube was filled with 20 mL of Mueller Hinton Broth (MHB), and a sterile cotton swab was used to roll on the GV colonies on the agar plate and transferred into the MHB to make the inoculum. The concentration of bacteria per mL of the inoculum was determined using a spectrophotometer. The inoculum was then centrifuged, and the bacteria were pelleted down. The supernatant was discarded, and the remaining pellet in the 50 mL centrifuge tube was mixed with 2 mL HBSS (Gibco, Massachusetts, United States). A concentration of 10^2^ CFU/mL of GV suspended in HBSS was used for intrauterine inoculation.

In optimizing the dose of inoculum, the lower dose of 10^2^ CFU/mL of GV was used based upon our preliminary results that showed intrauterine inoculation with a higher concentration of GV (10^3^ and 10^4^ CFU/mL) brought about unacceptably high mortality for both the dams and fetuses.

The volume for intraamniotic injection was 0.5 mL, as a volume of more than 0.5 mL resulted in a higher incidence of leakage, resulting in sepsis and mortality to the dams.

### Tissue Processing for Histomorphometry and Histopathology

The placenta was fixed in 4% paraformaldehyde (PFA4%) solution, dehydrated, and embedded in paraffin wax. The placenta tissues were sectioned (3–5 μm thickness) and stained using a standard hematoxylin and eosin (H&E) protocol. The slides were evaluated for the inflammatory infiltrates, edema, and necrosis. Aggregation of syncytial nuclei forming multinucleated cells was observed and quantified by counting the number of cells in five different high power fields (HPF; 400x) for each specimen. The average number per HPF was then calculated. An additional macrophage marker antibody MAC387 (Abcam, Cambridge, United Kingdom) staining was performed.

For fetal lung fixation, a 50 cm^3^ syringe was filled with 30 mL of chilled PFA4%, and the lungs were placed carefully into a syringe after first removing the plunger. The plunger was then reinserted, excess air expelled and the syringe tip occluded with a stopper. The syringe plunger was drawn with about 15 pulls to create a vacuum to inflate the alveoli for better lung fixation. The tissue was rinsed, dehydrated and embedded in paraffin wax. The lung tissue was then sectioned and stained by routine H&E staining protocol.

The lung morphometry assessment was carried out to assess the number of alveoli, alveolar surface area, and the thickness of alveolar septae. Five random non-overlapping HPF were viewed and measurements taken for each tissue section. Firstly, the number of alveoli was recorded. The alveolar area and the alveolar septal thickness were assessed using the DP2-BSW software (Olympus Corporation, Tokyo, Japan). The average alveolar surface area was determined by the sum of all closed areas, divided by the number of alveoli. The mean alveolar septal thickness was calculated from the average of three thickest septae.

### Immunohistochemical Staining of the Lung Tissue

Lung tissues were sectioned at 4 μm thickness and stained with mouse monoclonal Ki-67 antibody (Dako, Glostrup, Denmark) diluted 1:200. The DAKO ARK™ (Animal Research Kit) (Dako, California, United States) detection was used according to the manufacturer's instructions. Briefly, the sections were deparaffinized and dehydrated with xylene and graded alcohols. Antigen retrieval was carried out by heating for 30 min at 110°C in Tris-EDTA solution pH 9, followed by peroxidase blocking to quench endogenous peroxidase. Sections were then incubated for 15 min with the prepared biotinylated primary antibody, followed by streptavidin-peroxidase incubation for 15 min. Finally, 3,3′Diaminobenzidine (DAB) substrate chromogen was applied onto the sections for 5 min and counterstained with Mayer's Hematoxylin solution.

Three random fields of each lung tissue section were image-captured using high magnification (400×) with a B×40 microscope (Olympus Corporation, Tokyo, Japan). Two hundred nuclei in each field were counted manually, and a total of 600 nuclei per tissue section were analyzed. An average percentage of positively stained cells for every 100 nuclei were calculated as the proliferation index.

### Statistical Analysis

The Shapiro–Wilk test was applied to assess the normality of data. The mean differences between groups were compared with an unpaired two-tailed Student's *t*-test. The results were expressed as mean ± SD. All statistical analyses were performed with SPSS 22.0 (PASW Statistics, Chicago, USA). The *p*-values of < 0.05 were considered to be statistically significant.

## Results

### Animal Survival and Clearance of GV in Amniotic Fluid After 7 Days of Inoculation

In the experiment, all dams and their fetuses were alive at day 28 of gestation. There was neither preterm labor nor complications of the surgical procedure observed. Of note, no GV was cultured from the amniotic fluid of any of these fetuses after 7 days of inoculation. In contrast, all the bacterial cultures used to inoculate in the preceding 7 days when back-cultured were confirmed positive and yielded 10^2^ CFU/mL of GV in each sample.

### GV Infection Associated With Decreased Gross Body, Lung, and Placenta Weights

The weight of fetuses in the GV was significantly lower as compared to their littermate controls (mean gross weight, 19.8 ± 3.8 g vs. 27.9 ± 1.7 g, respectively, *p* < 0.001). The fetal lung weight was also significantly lower in the GV as compared with their littermate controls (mean fetal lung weight, 0.59 ± 0.11 g vs. 0.91 ± 0.08 g, respectively; *p* = 0.002). The placenta weight in GV infection was significantly lower as compared to the controls (mean placenta weight, 5.5 ± 1.0 g vs. 6.5 ± 0.7 g, respectively, *p* = 0.027) (Table 1).

**Table 1:** Comparison of the gross body, lung weights of fetuses, and the placental weights in gram (g) between saline-inoculated and GV*-*inoculated pregnant rabbits.

**Weights, g**		**Saline (*n* =9)**	**GV (*n* = 12)**	***p*-value**
Fetuses Gross body	Dam 1	30.0 28.0 28.0 27.0	14.0 22.0 21.0 22.0	
	Dam 2	25.0 28.0 26.0	15.0 16.0 23.0 21.0	
	Dam 3	30.0 29.0	22.0 15.0 26.0 21.0	
Mean gross body (SD)		27.9 (1.7)	19.8 (3.8)	<0.001
Fetuses Lung	Dam 1	1.00 0.82 1.00 0.92	0.60 0.70 0.60 0.61	
	Dam 2	0.81 0.90 1.00	0.45 0.63 0.72 0.60	
	Dam 3	0.90 0.85	0.55 0.42 0.81 0.50	
Mean fetal lung (SD)		0.91 (0.08)	0.59 (0.11)	0.002
Placenta,	Dam 1	8.0 6.0 6.5 6.0	4.5 5.5 7.0 7.5	
	Dam 2	6.8 6.0 6.5	5.0 4.5 5.5 5.0	
	Dam 3	6.5 7.0	5.5 4.3 6.5 5.5	
Mean placenta (SD)		6.5 (0.7)	5.5 (1.0)	0.027

*GV, Gardnerella vaginalis*.

### GV Infection Increases Multinucleated Syncytiotrophoblasts in the Placenta

**Figure 2:**
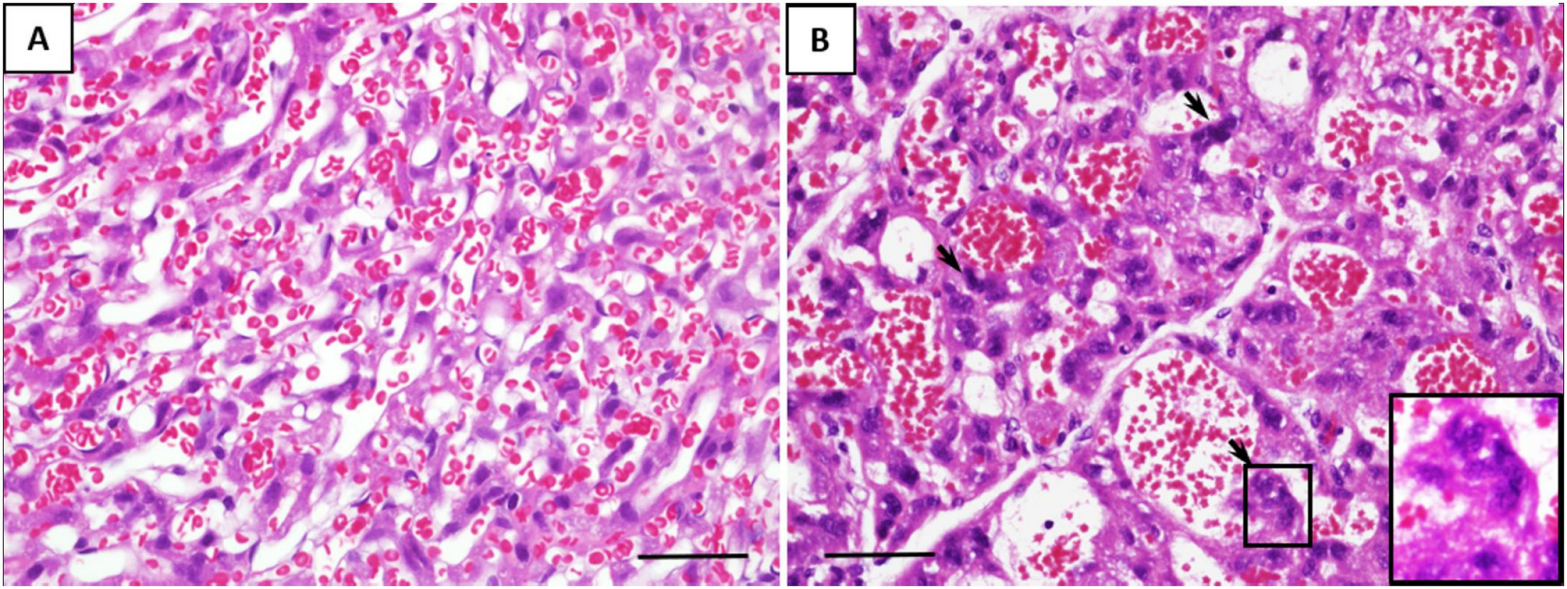
Representative histological changes of the placenta from **(A)** saline-inoculated and **(B)** GV-inoculated groups. GV exposed cases showed dilated vascular spaces and a significant increase in multinucleated syncytiotrophoblasts (arrows). These cells stained negative for the macrophage marker MAC387 (scale bar = 100 μm).

Microscopically, the histological characteristics of inflammation were not evident in the placenta tissues. However, the number of multinucleated syncytiotrophoblasts per HPF was two-fold higher in the placenta of fetuses exposed to GV than those unexposed (mean cell count per HPF, 83 ± 15 vs. 42 ± 13, respectively, *p* < 0.001). These cells display condensed nuclei without prominent nucleoli, and the surface macrophage marker, MAC387, were negative, together these are characteristics of the multinucleated syncytiotrophoblasts, also present and usually recognized as syncytial knots in the human equivalent (Figure 2).

### GV Infection Increases the Alveolar Septal Thickness and Proliferative Index in the Lung Interstitium

**Figure 3:**
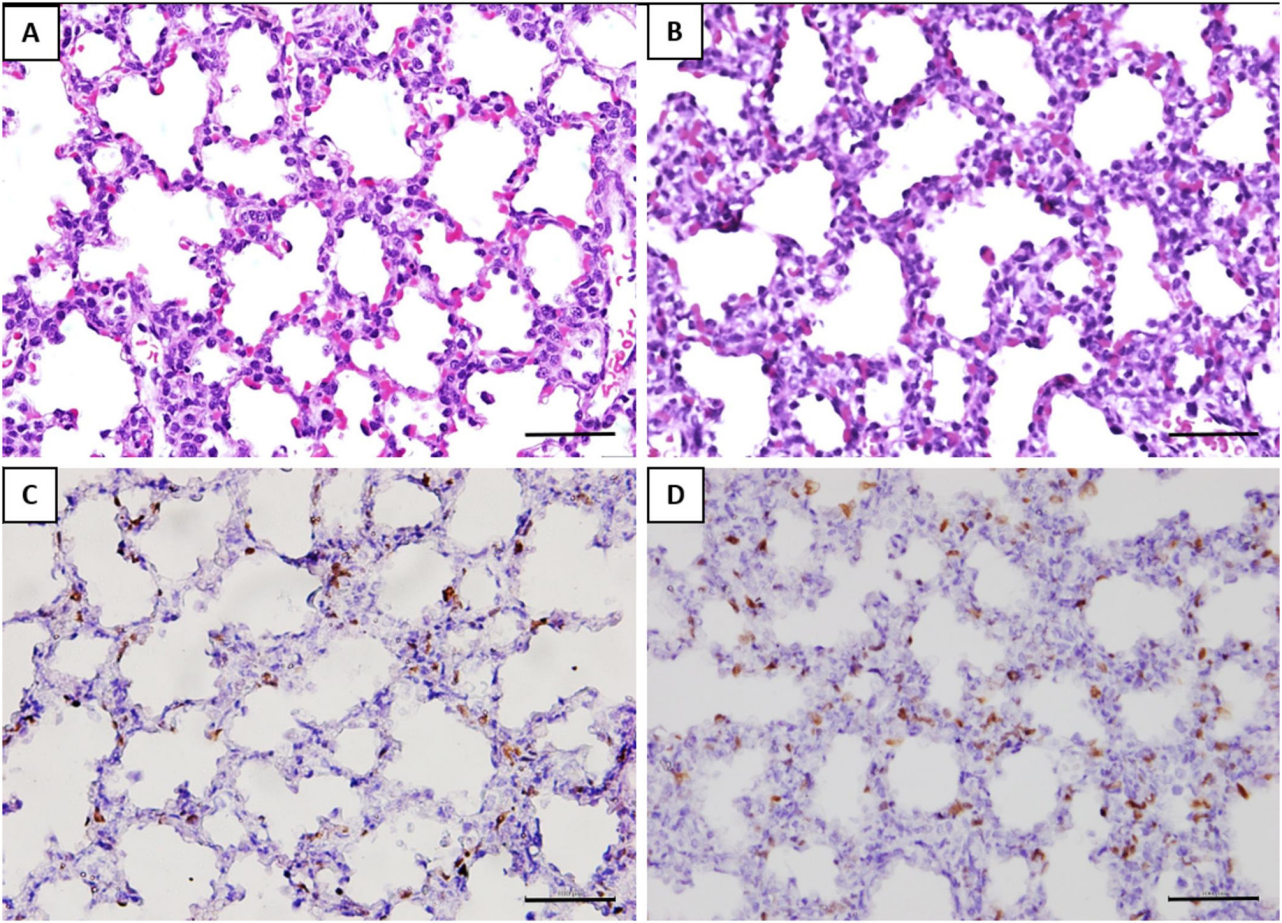
Histomorphometry and immunostaining of the fetal lung with or without exposure to GV. H&E staining of **(A)** saline-inoculated group **(B)** GV*-*inoculated group. The alveolar septae in the GV-inoculated group were thicker as compared to the saline-inoculated group (scale bar = 100 μm). There was no inflammatory cell infiltration observed in the alveoli or lung interstitium in both groups. Immunohistochemical staining showed significantly more Ki-67 positive cells in the interalveolar septae of fetal lungs in the **(D)** GV-inoculated group than **(C)** saline-inoculated group (scale bar = 100 μm).

The alveolar septa measured in the fetuses of the GV infected was approximately 1.2-fold thicker than the control group (Table 2). Ki-67 immunohistochemical study revealed that the proliferative index (PI) in the interalveolar septum was 1.8-fold higher in the GV than the control group (Table 2). The lung histology and immunohistochemical staining of Ki-67 antibody were illustrated in Figure 3.

**Table 2:** Comparison of the fetal lung histomorphometry indices between saline-inoculated and GV-inoculated pregnant rabbits.

**Lung indices**	**Saline**	**GV**	***p*-value**
Alveoli number, n^*^	15 (3)	14 (3)	0.260
Alveolar surface area, μm^2^	1,792.1 (1,006.2)	1,806.0 (1,060.7)	0.800
Alveolar septa thickness, μm	12.4 (3.8)	14.8 (2.8)	0.007
Interstitium—Proliferative index, %	14.2 (2.9)	24.9 (6.6)	0.011

* *This number was the average of the total number of alveoli counted in five random non-overlapping high power field (400×)*.

*Values are expressed as mean (SD)*.

*GV, Gardnerella vaginalis*.

The histology of lung tissue revealed no alveolar or interstitial inflammation in the GV and control group. The total number of alveoli and their total surface area did not vary between groups (Table 2).

## Discussion

In this study, we demonstrated that intrauterine GV infection was associated with features indicative of fetal growth restriction, lower gross body, lung, and placenta weights. This is consistent with previous studies on GV infection during pregnancy (36, 37). We found the fetal growth restriction was associated with increased multinucleated cells in the placenta of the GV-inoculated group, which has not been previously reported. These multinucleated cells also exhibited condensed nuclei and absent nucleoli which appear similar to syncytial knots (SK) in the human placenta. They are pathognomonic of syncytiotrophoblast apoptosis (38, 39), and are present in cases of infants born as IUGR (19, 20, 38). How SKs are formed are still not clearly understood (40). Histologically, they display condensed nuclei, no nucleoli, and devoid of transcriptional activity (41–43). SKs are generally an indicator of placenta aging, and an excessive number of SKs is suggestive of uteroplacental malperfusion (40, 44). The presence of multinucleated cells resembling SK had also been previously reported in the rabbit model of toxemia (45).

We speculate with infection, the direct insult to the trophoblasts could be mediated by the Toll-like Receptor-2 (TLR-2), which is expressed on the trophoblastic membrane (46, 47). TLR-2 recognizes the bacterial peptidoglycan and lipoteichoic acid, which are present on the bacterial wall of GV (47, 48). The ligation of TLR-2 activates MyD88/FADD-dependent caspases and/or NF-kappaB signaling pathways, which lead to cell death (49, 50) and the inflammation cascade. Moreover, *in vitro* peptidoglycan treatment on human trophoblastic cells resulted in a dose-dependent increase in cell apoptosis (50). The next phase of our study will include determination of the inter-related mechanisms underlying these observations.

In addition, intrauterine infection may affect placenta vasculogenesis (51–53). Altered placental vascular re-modeling, causing reduced perfusion and oxygen transport across the feto-maternal interface were observed in animal studies (54, 55). The dysregulation of several angiogenic mediators accompanied by uteroplacental malperfusion leads to hypoxia and adverse pregnancy outcomes (52, 56–58). The hypoxia-inducible factor 1α (HIF-1α) mediates cell apoptosis (59, 60), transcribes upregulation of anti-angiogenic mediators, e.g., sFlt-1 (61), that were increased in the placenta of IUGR infants (62, 63).

The increased SKs observed with GV infection implicates trophoblastic dysfunction and apoptosis, reducing the functional mass of the placenta, thus limiting nutrient transport and restricting intrauterine fetal growth (64). Even so, Burton et al. (40) reported that multinucleated syncytiotrophoblasts, important in placental development, may consist of sprouts. The increased numbers of these cells on the other hand may thus signify trophoblastic proliferative response to an increased need from the insult inflicted by GV.

We noted that there was no viable GV recovered from the amniotic fluid after 7 days of inoculation. The possible explanation could be clearance of the organism by the host immune response. In McDuffie et al. (37) study on chronic chorioamnionitis, a decreasing trend in the amniotic fluid positive cultures of GV from day four through six after inoculation, may support the role of host immune response in eradicating the organisms from the amniotic cavity with time. Additionally, our preliminary experiments with a higher dose of GV led to an increased demise of the dams and rates of abortion. Two earlier studies had used the intrauterine infection model to investigate the impact of higher doses of GV during pregnancy. Field et al. (36) endoscopically inoculated a relatively high concentration of GV into the uterine horns of pregnant rabbits and reported a significantly lower live birth rate (80 vs. 90%), lower live birthweight (12.82 g vs. 16.82 g) and more neuronal necrosis (60 vs. 0%) in GV-inoculated rabbits as compared to the saline-inoculated control. Nearly a decade later, McDuffie et al. (37) used a similar animal model and found a positive association between the duration of infection with the amniotic fluid proinflammatory cytokine (TNF-α) levels and fetal brain histological index scores. In both studies, high dose GV resulted in 20% abortion and 10% preterm labor (31, 36, 65).

In using a relatively lower concentration of inoculum but injected direct intra-amniotically, our study produced a 100% survival rate without any preterm birth or spontaneous abortion. A lower dose of GV at 0.5 × 10^2^ CFU, although eventually cleared by the host immune system, could have exerted injury to the fetal organs in the process (66). Changes to the placenta have been discussed above, but we also observed effects on the developing fetal lung. The lungs of low dose GV-infected fetuses demonstrated a significant degree of septal thickening as a result of increased cellular proliferation in the lung interstitium. Several preclinical models of intrauterine infection with low dose pathogens also yielded similar lung histopathology, which resembled the features of BPD in humans (24–26). Intrauterine infection has been shown to induce cellular proliferation via the effects of proinflammatory cytokine. Interleukin 1 beta (IL-1beta) is one of the central cytokines which has been implicated in the pathogenesis of BPD (67–70). The lung of IL-1beta-expressing gene-targeted mice revealed increased alveolar septal thickness accompanied with abnormal deposition of elastin in the interstitium (70) disrupting the lung morphogenesis. Moreover, prophylactic subcutaneous injection of IL-1 receptor antagonists (IL-1Ra) has been shown to prevent the BPD-like lung changes in the mice exposed to intrauterine LPS and postnatal hyperoxia (71). Our results showed unremarkable alteration in the number of alveoli and the total alveolar surface area with GV infection that are features of alveolar simplification reported in the “new BPD” (72). Future studies sufficiently powered with the appropriate sample size are necessary to confirm if these findings are induced with GV infection.

Antigen Ki-67 is a nuclear protein that is associated with cellular proliferation, and as such any cellular mitosis will display a high number of Ki-67. In this study, we demonstrated an increase in the proliferative cell index in the lung interstitium. Lung interstitial cells are derived from the mesenchyme and play a crucial role in alveolarization, along with the epithelium (73). Myofibroblast is the major interstitial cell that is critically involved in secondary septation and alveolarization. The dysregulation of myofibroblast has been implicated in the development of BPD (73, 74). Myofibroblasts markedly decreased after the alveolarization phase, and their persistent high numbers is associated with fibrotic lung diseases (75, 76). The regulatory pathways of myofibroblast during the different phases of lung development are still unclear. We are pursuing further studies to determine if lower dose of GV infection induced an increased interstitial lung myofibroblast activity resulting in alveolar septal hypertrophy. This may potentially impede alveolar gaseous exchange and predispose to pulmonary hypertension, features commonly associated with BPD.

Future studies with this model are in the planning to determine the signaling pathways involved leading to the multinucleated syncytiotrophoblasts formation and alveolar septal hypertrophy. Specific cell staining for alveolar type I, II pneumocytes, interstitial myofibroblasts, vascular bundles, and extracellular matrix composition could provide additional insights to the current understanding on the pathogenicity of intrauterine GV infection in modifying fetal lung growth. It would also be valuable to study the postnatal functional outcomes of these fetuses with abnormal growth and lung development.

## Conclusion

In conclusion, low dose intrauterine GV infection leads to fetal growth restriction, increased placenta multinucleated syncytiotrophoblasts, and disrupted lung morphogenesis with alveolar septal hypertrophy. These findings may lead to studies in human pregnancies to determine if antenatal GV infection with and without treatment similarly results in fetal growth restriction and alveolar septal re-modeling that predisposes to BPD development.

## Data Availability Statement

The original contributions presented in the study are included in the article/supplementary materials, further inquiries can be directed to the corresponding author/s.

## Ethics Statement

The animal study was reviewed and approved by Universiti Kebangsaan Malaysia Animal Ethics Committee (UKMAEC).

## Author Contributions

F-CC designed and created the animal model and was the principal investigator of the study. CL coordinated the breeding, rearing of the rabbits, fine-tuning of the model, and executed majority of the animal experiments. All authors have made a substantial contribution to the research work and manuscript preparation and reviewed and approved the final version of the manuscript.

## Conflict of Interest

The authors declare that the research was conducted in the absence of any commercial or financial relationships that could be construed as a potential conflict of interest.
